# Editorial: Physiological and pathological aspects of GnRH neuron system development

**DOI:** 10.3389/fendo.2023.1268663

**Published:** 2023-08-17

**Authors:** Konstantina Chachlaki, Roberto Oleari

**Affiliations:** ^1^Univ. Lille, Inserm, CHU Lille, Laboratory of Development and Plasticity of the Neuroendocrine Brain, Lille Neuroscience and Cognition, UMR-S 1172, Lille, France; ^2^FHU 1000 Days for Health, School of Medicine, Lille, France; ^3^University Research Institute of Child Health and Precision Medicine, “Aghia Sophia” Children’s Hospital, National and Kapodistrian University of Athens, Athens, Greece; ^4^Department of Pharmacological and Biomolecular Sciences, University of Milan, Milan, Italy

**Keywords:** GnRH neuron, congenital hypogonadotropic hypogonadism, kisspeptin neuron, glial cells, tanycyte, PCOS (polycystic ovarian syndrome)

The development of the Gonadotropin-releasing hormone (GnRH)-neuron system is a fascinating and complex process that plays a crucial role in the regulation of reproductive function. These hypothalamic neuroendocrine neurons control pubertal onset and fertility in most vertebrates through the strictly regulated release of the GnRH peptide, consisting of pulses and surges. Interestingly, GnRH-neurons developmentally originate outside the brain, from stem cells located in the nasal placode. During embryogenesis, the GnRH-neurons migrate along the axons of the terminal nerve to arrive at the hypothalamus, and this migration also relies on the correct formation of olfactory ensheathing cells (OECs), an olfactory system-specific glial population that migrate with the GnRH-neurons (1).

After the migration of the GnRH-neurons and in early postnatal life, they undergo alterations in their activity, biosynthetic profile, and connectivity, including axon elongation to the median eminence. The development of the GnRH neuron system is a highly dynamic process that involves multiple stages and a complex interplay of genetic, epigenetic, environmental, and hormonal factors. Unraveling the contributions and interactions of GnRH-neurons with other cells and signaling molecules, as well as dissecting the precise timing and sequence of events during GnRH neuron development is a major challenge in understanding the physiopathological aspects of GnRH neuron system development.

Despite these challenges, ongoing research efforts, including the use of advanced technologies and improved animal models, are gradually improving our understanding of the physiopathological aspects of GnRH neuron system development. The integration of multiple disciplines, such as developmental biology, genetics, neurobiology, and endocrinology, is crucial for making progress in this field. In this editorial, we will explore the physiological and pathological aspects of GnRH neuron system development, shedding light on the intricacies of this fascinating field. Join us as we delve into the latest research and gain a deeper understanding of the mechanisms that underlie this critical system.

An editorial on GnRH neuron physiology would be incomplete without acknowledging the pivotal role of kisspeptin research in unraveling the intricate mechanisms underlying the regulation of reproductive processes in mammals, beautifully reviewed from Xie et al. Understanding the influence of kisspeptin on the reproductive axis and related mechanisms could have important implications for disease diagnosis and treatment.

To this aim, the study from Shen et al. employ optogenetics to selectively stimulate Kisspeptin neurons in the arcuate nucleus (ARH) and anteroventral periventricular nucleus (AVPV) in order to investigate their role in the luteinizing hormone (LH) surge. The authors also examine the effects of natural steroid hormone milieu and the release of glutamatergic neurotransmitters on LH secretion. This study adds new insights into the mechanisms underlying the LH surge and may potentially contribute to improving reproductive outcomes in mammals. Indeed, altered responses to kisspeptinergic neurotransmitter systems may directly impact reproductive physiology. The study from Bhattarai et al., by examining the response of various neurotransmitters and neurosteroids regulating GnRH neuronal activities between letrozole-induced PCOS and normal mice, provides new insights into the possible neuroendocrine disruptions in PCOS at the GnRH level. The authors conclude that PCOS may directly affect the neurotransmitter system regulating GnRH activity at the hypothalamic level, thus opening up new avenues for therapeutic targets for PCOS.

The microenvironment of GnRH neurons participates in the modulation of their activity, particularly in the synthesis and secretion of GnRH. Tanycytes are able to form self-renewing neurospheres and produce primarily astrocytes, and their removal may modify the glial microenvironment around the GnRH neurons and fibers. Here, *Dr* Butruille et al. investigate the role of glial fibrillary acidic protein (GFAP)-expressing tanycytes in the male reproductive function and their impact on the GnRH system within the hypothalamus. The findings of this study support the view that GFAP-positive tanycytes play a critical role in the regulation of male reproductive function. The depletion of these cells alters hormone levels, testicular weight, and sexual behavior, in addition to causing significant structural alterations that may hamper the tanycyte-to-GnRH-neuron communication processes controlling reproduction. The study provides new insights into the functional and morphological interactions between GFAP-expressing tanycytes and GnRH axon terminals in the hypothalamus, highlighting their central role in maintaining the neuroendocrine system.

Overall, understanding the maintenance of the postnatal GnRH system for the treatment of fertility issues in humans and other animals could be of major importance since disruption of processes responsible for the initiation and maintenance of GnRH neuronal identity can lead to reproductive disruption similar to the congenital hypogonadotropic hypogonadism (CHH) phenotype. In their review article, Chung et al. emphasize the need to focus on how GnRH neurons initiate and maintain their identity during prenatal and postnatal periods, addressing two questions: can a disease state such as CHH lead to the postnatal loss of fully differentiated GnRH neurons, and can the postnatal GnRH neuronal loss be reversed by beneficial cues?

In conclusion, this Research Topic summarizes and discusses the most recent findings about the intricate biological networks underlying GnRH neuron system, with a specific highlight on kisspeptin neurons and tanycytes that are increasingly emerging as fundamental for the correct functioning of GnRH neurons, and without neglecting a glance towards novel therapeutic targets of GnRH neuron-related disorders.

## Author contributions

RO: Writing – review & editing. KC: Writing – original draft.

## References

[1] Duittoz AH Forni PE Giacobini P Golan M Mollard P Negrón AL. Development of the gonadotropin-releasing hormone system. J Neuroendocrinol (2022) 34(5):e13087. doi: 10.1111/jne.13087 35067985 PMC9286803

